# Contribution of Nlrp3 Inflammasome Activation Mediated by Suilysin to Streptococcal Toxic Shock-like Syndrome

**DOI:** 10.3389/fmicb.2020.01788

**Published:** 2020-08-14

**Authors:** Liqiong Song, Xianping Li, Yuchun Xiao, Yuanming Huang, Yongqiang Jiang, Guangxun Meng, Zhihong Ren

**Affiliations:** ^1^State Key Laboratory for Infectious Disease Prevention and Control, National Institute for Communicable Disease Control and Prevention, Collaborative Innovation Center for Diagnosis and Treatment of Infectious Diseases, Chinese Center for Disease Control and Prevention, Beijing, China; ^2^Research Units of Discovery of Unknown Bacteria and Function (2018 RU010), Chinese Academy of Medical Sciences, Beijing, China; ^3^State Key Laboratory of Pathogen and Biosecurity, Institute of Microbiology and Epidemiology, Academy of Military Medical Sciences, Beijing, China; ^4^The Center for Microbes, Development and Health, CAS Key Laboratory of Molecular Virology and Immunology, Institut Pasteur of Shanghai, Chinese Academy of Sciences, University of Chinese Academy of Sciences, Shanghai, China

**Keywords:** *Streptococcus suis*, suilysin, interlukin-1β, inflammasome, caspase-1

## Abstract

**Objective**: The aim of this study was to investigate the molecular mechanism of inflammasome activation in response to *Streptococcus suis* serotype 2 (SS2) infection and its contribution to the development of streptococcal toxic shock-like syndrome (STSS).

**Methods**: To verify the role of suilysin (SLY) in STSS, we infected bone-marrow-derived macrophages (BMDMs) *in vitro* and C57BL/6J mice intraperitoneally (IP) with the SS2 wild-type (WT) strain or isogenic *sly* mutant (∆SLY) to measure the interleukin (IL)-1β release and survival rate. To determine the role of inflammasome activation and pyroptosis in STSS, we infected BMDMs from WT and various deficient mice, including *Nlrp3*-deficient (Nlrp3^−/−^), *Nlrc4*-deficient (Nlrc4^−/−^), *Asc*-deficient (Asc^−/−^), *Aim2*-deficient (Aim2^−/−^), *Caspase*-1/11-deficient (Caspase-1/11^−/−^), and *Gsdmd*-deficient (Gsdmd^−/−^) *ex vivo*, and IP injected WT, Nlrp3^−/−^, Caspase-1/11^−/−^, and Gsdmd^−/−^ mice with SS2, to compare the IL-1β releases and survival rate *in vivo*.

**Results**: The SS2-induced IL-1β production in mouse macrophages is mediated by SLY *ex vivo*. The survival rate of WT mice infected with SS2 was significantly lower than that of mice infected with the ∆SLY strain *in vivo*. Furthermore, SS2-triggered IL-1β releases, and the cytotoxicity in the BMDMs required the activation of the NOD-Like Receptors Family Pyrin Domain Containing 3 (Nlrp3), Caspase-1/11, and gasdermin D (Gsdmd) inflammasomes, but not the Nlrc4 and Aim2 inflammasomes *ex vivo*. The IL-1β production and survival rate of WT mice infected with SS2 were significantly lower than those of the Nlrp3^−/−^, Caspase-1/11^−/−^, and Gsdmd^−/−^ mice *in vivo*. Finally, the inhibitor of the Nlrp3 inflammasome could reduce the IL-1β release and cytotoxicity of SS2-infected macrophages *ex vivo* and protect SS2-infected mice from death *in vivo*.

**Conclusion**: Nlrp3 inflammasome activation triggered by SLY in macrophages played an important role in the pathogenesis of STSS.

## Introduction

*Streptococcus suis* is a common swine pathogen, which not only results in a great loss to swine industry every year but also causes meningitis and streptococcal toxic shock-like syndrome (STSS) in humans (Ye et al., 2006; Yu et al., 2006; Gottschalk et al., 2007; Huong et al., 2014). More than 30 serotypes of *S. suis* have been identified (King et al., 2001), among which serotype 2 is considered as the most common and virulent. *Streptococcus suis* serotype 2 (SS2) is further divided into four predominant sequence types using multilocus sequence typing, including sequence types 1, 7, 25, and 28 (Fittipaldi et al., 2011). Among patients infected with strain ST7 of *S. suis*, the incidence of STSS is significantly higher than that among patients infected with the other sequence type of SS2. ST7 is thought to have been responsible for two large outbreaks of human SS2 infections in China in 1998 and 2005 (Hu et al., 2000; Ye et al., 2006; Yu et al., 2006). The serum proinflammatory cytokines in STSS patients, especially interleukin 6 (IL-6), IL-1β, and tumor necrosis factor (TNF), gamma interferon (IFN-ɣ), IL-12, and monocyte chemoattractant protein 1 (MCP-1), are significantly higher than those in meningitis patients (Ye et al., 2009). It was generally accepted that the severe outcome of the SS2 infection was closely related to the excess innate immune response of the host. However, how the overproduction of proinflammatory cytokines occurs in STSS is not fully understood.

A variety of virulence factors participate in the *S. suis* pathogenesis, for example, the capsular polysaccharide and lipoprotein (LP). Among these virulence factors, suilysin (SLY) was found to be closely related to bacterial dissemination and host inflammation (Lun et al., 2003). SLY, an extracellular protein secreted by SS2, belongs to a family of cholesterol-dependent cytolysins, including listeriolysin O of *Listeria monocytogenes* and pneumolysin of *Streptococcus pneumonia*, which are the multifunctional proteins responsible for hemolytic activity, apoptosis, and cytokine-inducing activity (Kim et al., 2010; Witzenrath et al., 2011). Notably, a highly virulent strain (ST7) that causes a more severe invasive infection can produce a greater amount of SLYs than non-epidemic strains (He et al., 2014). SLY-positive strains have also been reported to result in more severe symptoms than SLY-negative strains (Jacobs et al., 1996; He et al., 2014). Moreover, it was reported that SLY could stimulate the host immune system to produce massive amounts of proinflammatory cytokines, for example, IL-1β, IL-6, and TNF-α (Lun et al., 2003; Tenenbaum et al., 2016). The vaccine containing purified SLY derived from SS2 had immuno-protective effects against SS2-induced STSS for swine and mice due to its reduction of the proinflammatory response during SS2 infection (Jacobs et al., 1996). Furthermore, passive immunization using anti-SLY antisera protected mice from acute death after infection with SS2 and significantly reduced levels of proinflammatory cytokines. Therefore, it is necessary to further explore the role of SLY in the pathogenesis of SS2 infection.

Lavagna et al. (2019) reported that the IL-1β release plays a protective role during SS2 systemic infection by activating various inflammasomes, thus promoting host survival. The inflammasome is commonly composed of NOD-like receptors (NLRs) and the adapter molecule Asc. Multiple inflammasomes are involved in the host defense responses against various pathogens. The activated inflammasome can recruit and activate proinflammatory protease, Caspase-1. The activated form of Caspase-1 then cleaves the precursors of some subsequent cytokines (pro-IL-1β and pro-IL-18) into their corresponding mature forms, and it cleaves gasdermin D (Gsdmd) to remove its auto-inhibition. The N-terminal of Gsdmd generated by the cleavage induces cell pyroptosis, a programmed cell necrosis accompanied by the activation of the inflammatory cytokines IL-1β and IL-18, and forms pores on the cell membrane for IL-1β secretion (Shi et al., 2017). However, existing studies have not revealed the comprehensive molecular mechanism for the inflammasome activation triggered by SLY of SS2 and have not clarified the role of Nlrp3 inflammasome activation and pyroptosis in the pathogenesis of SS2-induced STSS.

To investigate the issues mentioned above, we constructed a SLY-mutant strain and used mouse macrophages deficient in Nlrp3, Nlrc4, Aim2, Caspase-1/11, Asc, and Gsdmd to determine whether SLY mediates inflammasome activation and pyroptosis and explored the role of inflammasome activation and pyroptosis in SS2-induced STSS. Our findings demonstrate a critical role of SLY in activating inflammasomes during STSS; the deficiencies in the Nlrp3 or Nlrp3 inhibitors could protect SS2-infected mice from death.

## Materials and Methods

### Ethics Statement

This study was approved by the Laboratory Animal Welfare & Ethics Committee of the National Institute for Communicable Disease Control and Prevention, Chinese Center for Disease Prevention and Control. This study was carried out in accordance with the recommendations of the Animal Management Regulations (2017/03) and measures for the Ethical Review of Biomedical Research Involving Humans (2016/12) [SYXK (Beijing 2012-0022)].

All operations in our study are performed in the Biosafety Level II laboratory and Animal Biosafety Level II laboratory.

### Bacterial Culture and Preparation of the Concentrated Supernatant Protein (SS2-S/N and ∆SLY-S/N)

The wild-type (WT) SS2 strain 05ZYH33 (ST-7) and its isogenic *sly* mutant (∆SLY; He et al., 2014; Pian et al., 2015) were obtained from Yongqiang Jiang’s laboratory at the Institute of Microbiology and Epidemiology, Academy of Chinese Military Medical Sciences. These bacteria were cultured on goat blood agar at 37°C overnight. The isolated colonies were inoculated into Todd–Hewitt broth (BD, USA).

Bacterial culture supernatants of the SS2 WT or ∆SLY strain were centrifuged at 10,000 rpm for 10 min and then filtrated with a 0.22 μM filter. Then, both the cell-free supernatants were centrifuged using Amicon Ultra-4 10 K Centrifugal Filter Devices (Millipore, USA) and concentrated. The concentration of the supernatant protein containing SLY (SS2-S/N) or without SLY (∆SLY-S/N) was measured using a Pierce™ BCA protein kit (Pierce, USA).

### Mice and Cell Culture

All mice used in our experiments are on a C57BL/6 genetic background and all experiments were carried out with age and gender matched mice (8–10 weeks old, female). C57BL/6 WT mice were obtained from Beijing Vital River Laboratory Animal Technology Co. Ltd., *Asc*-deficient (Asc^−/−^) mice were provided by Vishva M. Dixit of Genentech, and *Nlrp3*-deficient (Nlrp3^−/−^) mice were provided by Warren Strober from NIH, *Aim2*-deficient (Aim2^−/−^) were obtained from Dr. Meng Guangxun’s lab (Mariathasan et al., 2004; Hornung et al., 2009; Meng et al., 2009; Mao et al., 2013), and Caspase-1^−/−^mice were obtained from the Jackson Laboratory and crossed onto the C57BL/6 genetic background for 10 generations. These mice are also deficient for functional Caspase-11 (Kayagaki et al., 2011). *Nlrc4*-deficient (Nlrc4^−/−^) and *Gsdmd*-deficient (Gsdmd^−/−^) mice on a C57BL/6 background were obtained from Feng Shao’s lab at the Beijing Institute of Life Sciences (Shi et al., 2015).

Mouse bone-marrow-derived macrophages (BMDMs) were isolated from the abovementioned mice and cultured as previously described (Song et al., 2015). Peritoneal macrophages (PMs) were collected from peritoneal lavage using the procedure utilized by Kumagai et al. (1979). The purity of the macrophages obtained was around 90%, which was assessed by a flow cytometer using the F4/80 antibody. Differentiation of the THP-1 human monocytic cell line was achieved after incubation for 48 h in the presence of 10 nM phorbolmyristate acetate (PMA, P8139). All of the cultured cells were grown in RPMI 1640 at a maximum density of 1 × 10^6^ cells/ml.

### BMDMs Infected With Bacteria (SS2 or ∆SLY) and Treated With Concentrated Supernatant Proteins (SS2-S/N or ∆SLY-S/N) *ex vivo*

BMDMs from WT mice or deficient mice (Asc^−/−^, Nlrc4^−/−^, Aim2^−/−^, Nlrp3^−/−^, Casp-1/11^−/−^, and Gsdmd^−/−^) were infected with the SS2 WT or ∆SLY strain *ex vivo* at a multiplicity of infection (MOI) of 1 for 16 h without lipopolysaccharide (LPS,100 ng/ml, L3012, Sigma) priming. BMDMs treated with phosphate buffer saline (PBS) or LPS plus adenosine triphosphate (ATP; 500 μM, A2383, Sigma) were used as the negative and positive control, respectively.

In parallel, BMDMs from the WT mice or deficient mice (Asc^−/−^, Nlrc4^−/−^, Aim2^−/−^, Nlrp3^−/−^, Casp-1/11^−/−^, and Gsdmd^−/−^) were pretreated with LPS priming (100 ng/ml) for 3 h and washed off with PBS. Then, the cell-free concentrated supernatant proteins SS2-S/N (containing SLY) or ∆SLY-S/N (without SLY) were incubated with the primed BMDMs in a 24-well plates at a concentration of 200 ng/ml for 16 h, respectively.

Both the unprimed BMDMs and LPS-primed BMDMs were pre-incubated with various inhibitors, including KCl (50 mM, PB0440), oxidized ATP (oATP, 500 μM, A6779), N-acetyl-L-cysteine (NAC; 20 mM, A7250), Nlrp3 inhibitor MCC950 (10 μM, S7809), and Caspase-1 inhibitor Z-YVAD-FMK (10 μM, A3707) at the indicated concentrations for 1 h. Then, these unprimed BMDMs were infected with the SS2 WT or ∆SLY strain, and LPS-primed BMDMs were treated with the concentrated supernatant proteins SS2-S/N or ∆SLY-S/N for 16 h *ex vivo*, as described above.

### Mice Infection *in vivo*

To observe the survival of mice from a high-dose infection, C57BL/6J WT mice (female, 18–20 g) and various deficient mice (Nlrp3^−/−^, Casp-1/11^−/−^, and Gsdmd^−/−^) were injected intraperitoneally (IP) with 2 × 10^9^ CFU SS2 or its mutant (∆SLY) strain in 200 μl PBS. The mice of the negative control group received the same volume of PBS. There were 10 mice in each group, and their survival was monitored every 2 h up to 24 h.

To detect the inflammatory response to a low dose of SS2 infection without death, BMDMs of C57BL/6J WT and various deficient mice (Nlrp3^−/−^, Casp-1/11^−/−^, and Gsdmd^−/−^) were injected IP with 2 × 10^8^ CFU SS2 in 200 μl PBS, and the negative control mice were treated with the same volume of PBS (six mice per group). Mice were sacrificed 24 h after injection. The peritoneal lavage fluid (PLF) was harvested by rinsing the peritoneal cavity with 500 μl PBS. The harvested PLF was used for the IL-1β detection by ELISA.

To determine the effect of the Nlrp3 inhibitor MCC950 on the survival and inflammatory response of SS2-infected mice *in vivo*, mice were injected IP with MCC950 (50 mg/kg, qd) for 2 days and then infected with 2 × 10^8^ CFU SS2 2 h after injection. Mice were sacrificed 24 h after infection, then the PLF was harvested for IL-1β detection by ELISA. Those mice infected with 2 × 10^9^ CFU SS2 were monitored for their survival for 24 h.

The WT mice group infected with SS2 was used as common positive control group in all *in vivo* experiments. The WT mice treated with PBS were used as shared negative control group in all *in vivo* experiments.

### Detection of Cytokines and Lactate Dehydrogenase

All the cultured supernatants mentioned above were collected at the indicated time in this study. Lactate dehydrogenase (LDH) was measured for the cytotoxicity assessment using the Cytotox96 assay kit (Promega, USA). Interlukin 1β (IL-1β) and IL-6 were tested using ELISA kits (BD Biosciences, USA).

### Western Blotting Analysis

The cultured supernatants and bacterial lysates were collected at the indicated time after treatment (Liu et al., 2015). Proteins in the supernatants were extracted using the methanol – chloroform approach, and the cell pellets were lysed with the RIPA Lysis buffer (89901, Thermo) supplemented with a protease inhibitor cocktail (Roche). Finally, all the protein pellets were mixed with the SDS loading buffer. Both of the obtained proteins were applied to detection for pro-IL-1β/IL-1β, and pro-Caspase-1/Caspase-1 by immunoblotting. β-actin was chosen as the positive control. The antibodies against IL-1β (sc-52012), Caspase-1 (sc-56036), β-actin (4967S), and the secondary antibodies (IRDye 800-labeled anti-rabbit IgG; 611-132-002) were obtained from Santa Cruz Biotechnology. The proteins were quantified using an Odyssey infrared imaging system (LI-COR, Lincoln, NE).

### Statistical Analysis

All continuous variables were represented as the means ± standard deviations. The unpaired 2-tailed Student’s *t* test was used to compare the differences between two groups. Differences among three or more groups were compared using ANOVA. The survivals of different groups of mice were plotted with Kaplan Meir and their comparison was performed using the log-rank method. A value of *p* < 0.05 was considered statistically significant. Statistical analysis was done using SPSS 17.0 (SPSS Inc., Chicago, IL).

## Results

### IL-1β Releases and Cytotoxicity Are Induced by SS2 *in vitro*

**Figure 1:**
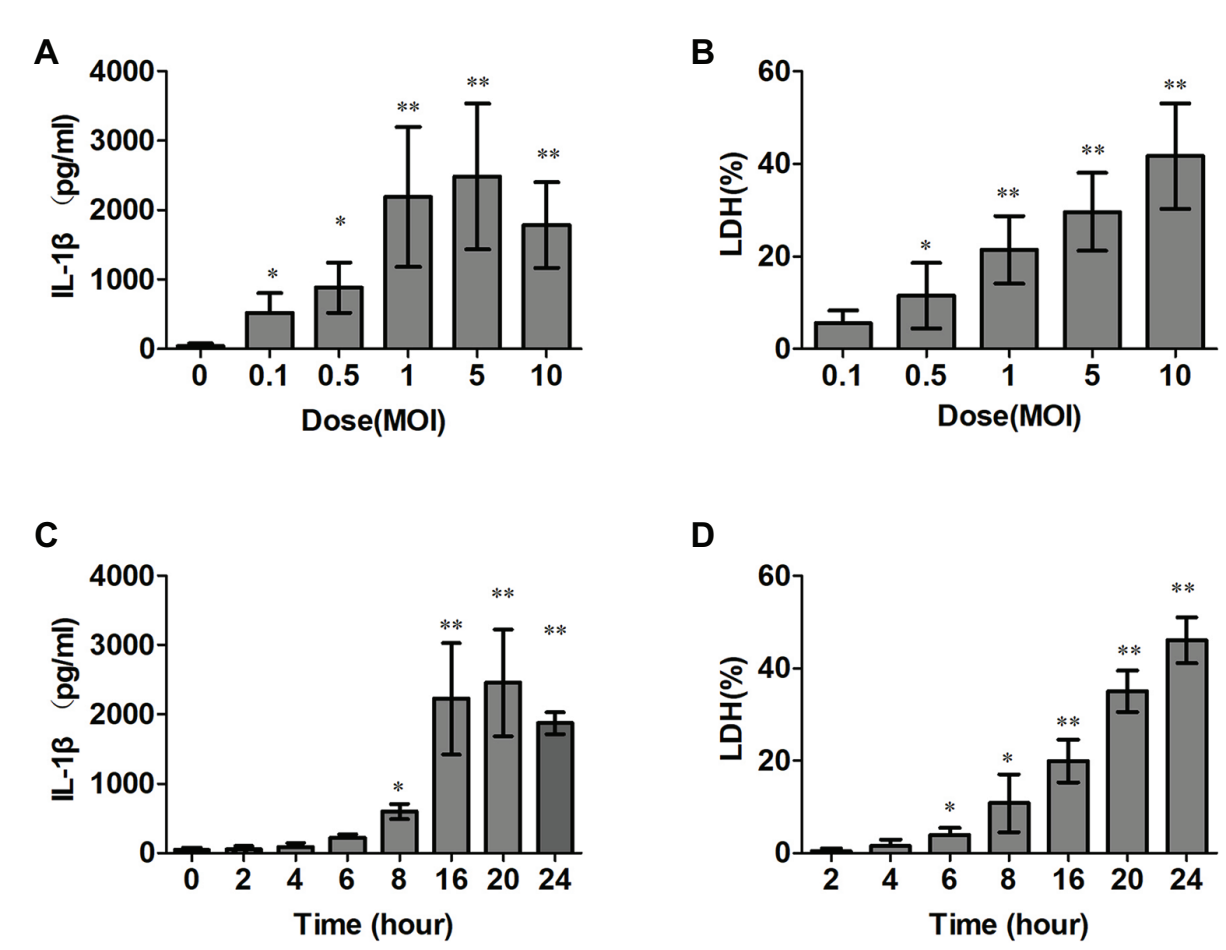
*Streptococcus suis* serotype 2 (SS2) trigged interleukin (IL)-1β production and induced cytotoxicity *in vitro*. (**A**,**B**) Bone marrow derived macrophages (BMDMs; 1 × 10^6^ cells/ml) were infected with SS2. The supernatants were harvested at different time points after infection. The IL-1β release was measured with ELISA **(A)**, and lactate dehydrogenase (LDH) leakage was detected using the CytoTox96® non-radioactive cytotoxicity kit **(B)**. (**C**,**D**) BMDMs were infected with SS2 at different doses, and the supernatants were harvested for IL-1β **(C)** and LDH detections **(D)** The data shown have the means ± standard deviations from three independent experiments. ^*^p < 0.05; ^**^p < 0.01.

To measure the kinetics of the IL-1β secretion and cytotoxicity induction by SS2 in macrophages, we infected mice PMs with SS2, as explained in the “Materials and Methods” section, and the data showed that SS2 gradually increased IL-1β and the cytotoxicity during the time course after the SS2 challenge. The optimal stimulation conditions for SS2 to induce IL-1β production was 16 h post infection and a MOI of 1 (Figures 1A,C) with peaked IL-1β production (2,200 ± 605 pg/ml) without too much cytotoxicity (20.2 ± 4.6%). Thus, this optimal condition was used for subsequent *ex vivo* experiments. Figures 1B,D show that SS2 induced cytotoxicity in a time‐ and dose-dependent manner.

### SLY Contributes to the IL-1β Release and Cytotoxicity of BMDMs *in vitro* and a Deficiency of SLY Protects Mice From Death *in vivo* in Response to the SS2 Infection

**Figure 2:**
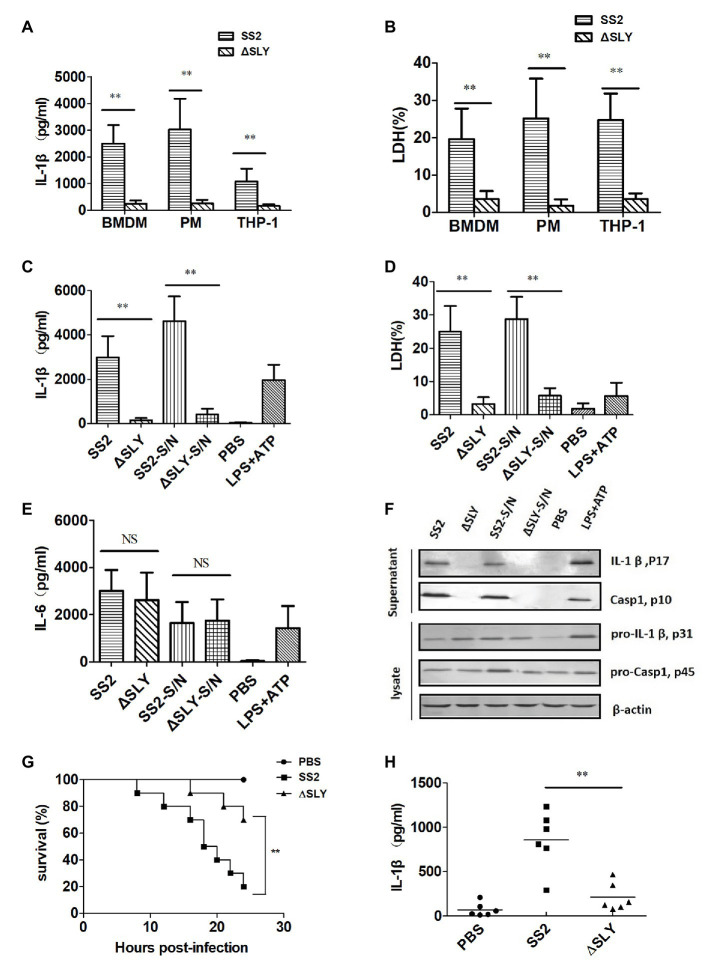
SS2-induced secretion of IL-1β is dependent on suilysin (SLY) *in vitro* and *in vivo*. (**A**,**B**) Three types of cells, including human monocytic THP-1 cells, mouse peritoneal macrophages (PMs), and BMDMs, were infected with SS2 or its SLY-deficient mutant (∆SLY); multiplicity of infection (MOI, 1) for 16 h. The supernatants were harvested for the measurement of IL-1β **(A)** and LDH **(B)**. **(C–E)** BMDMs (1 × 10^6^ cells/ml) were infected with SS2 or ∆SLY (MOI, 1) for 16 h and also treated in parallel with the concentrated supernatant proteins SS2-S/N (200 ng/ml) or ∆SLY-S/N (200 ng/ml) for 16 h after LPS priming for 3 h. Phosphate buffer saline (PBS) and LPS plus adenosine triphosphate (ATP) were used as the negative control and positive control, respectively. The supernatants were collected for IL-1β **(C)** and IL-6 **(E)** by ELISA and LDH assay **(D)**. **(F)** Immunoblotting was performed, and the culture supernatants were measured for IL-1β p17 and Caspase1 p10, and the cell lysates were analyzed for proIL-1β p31 and pro-Caspase1 p45. **(G)** 8-week-old C57BL/6J WT mice were injected intraperitoneally (IP) with a higher dose of SS2 or ∆SLY (2 × 10^9^ CFU/mouse in 200 μl PBS) and their survivals were observed every 2 h up to 24 h, with 10 mice in each group. The WT mice treated with SS2 and PBS were considered as the positive and negative control group, respectively. **(H)** 8-week-old WT C57BL/6J mice were injected IP with a lower dose of SS2 or ∆SLY (2 × 10^8^ CFU in 200 μl PBS). The WT mice treated with SS2 and PBS were considered as the positive and negative control group, respectively. These mice were sacrificed 24 h after injection. Their peritoneal lavage fluids (PLFs) were harvested by rinsing the peritoneal cavity with 500 μl PBS and measured for IL-1β by ELISA (six mice in each group). The data shown in panels **(A–E)** are the means ± standard deviations from three independent experiments. The data shown in panels **(F–H)** are from one of two independent experiments. ^*^p < 0.05; ^**^p < 0.01.

To determine which component of SS2 mediates the IL-1β secretion and cytotoxicity in multiple cells, PMs, BMDMs, and human THP-1 cells were infected with SS2 and its SLY-deficient isogenic mutant (∆SLY; MOI, 1). SS2 induced significantly more IL-1β and cytotoxicity compared with the ∆ SLY strain 16 h post infection (Figures 2A,B). To confirm whether the secreted SLY triggered the IL-1β and LDH release, we treated BMDMs with concentrated supernatant proteins (SS2-S/N containing SLY or ∆SLY-S/N without SLY). The SS2-S/N induced a significantly greater IL-1β and LDH release in BMDMs than ∆SLY-S/N did (Figures 2C,D). In contrast, the IL-6 secretions induced by different strains were comparable, suggesting that the production of IL-6 was not affected by SLY and was independent of the IL-1β release (Figure 2E). These results further confirmed that the SS2-induced-IL-1β secretion and cytotoxicity in the BMDMs were mediated by SLY.

To explore the molecular mechanisms of the SS2-induced IL-1β in BMDMs, we measured the amount of IL-1β (p17) and its immature precursor pro-IL-1β (p31) and the amount of Caspase-1 (p10) and its immature precursor pro-Caspase-1 (p45) in both supernatants and cell lysates using western blotting. The BMDMs were treated as indicated in the “Materials and Methods” section and LPS plus ATP was used as a positive control to stimulate the secretion of IL-1β. The data showed that all strains and concentrated supernatant proteins induced similar levels of immature pro-IL-1β in the cell lysates, but SS2 or SS2-S/N induced significantly more mature IL-1β in the supernatants than the ∆SLY strains or ∆SLY-S/N did. We also observed the presence of active Caspase-1 in the supernatants of the BMDMs treated with SS2 or SS2-S/N, but not in the supernatants of the cells treated with ∆SLY or ∆SLY-S/N (Figure 2F).

To verify the role of SLY in the pathogenicity of SS2 *in vivo*, we infected WT mice as noted in the “Materials and Methods” section and observed their activity and survival every 2 h until 24 h post infection. Eight out of ten WT mice infected with a higher dose of 2 × 10^9^ CFU SS2 strain died within 24 h, but only 2 out of 10 WT mice infected with 2 × 10^9^ CFU ∆SLY strains died within 24 h post infection (Figure 2G). To further evaluate whether SLY was involved in the IL-1β release in response to the SS2 infection *in vivo*, we injected IP WT mice with a lower dose of 2 × 10^8^ CFU SS2 or ∆SLY. Our results proved that the IL-1β production in the PLF from the SS2-infected WT mice was significantly higher than that in the ∆SLY-infected WT mice (Figure 2H). These findings suggested that SLY is a very important virulent factor.

### SS2-Triggered IL-1β Releases and Cytotoxicity Require the Activation of Caspase-1/11 and Gsdmd

**Figure 3:**
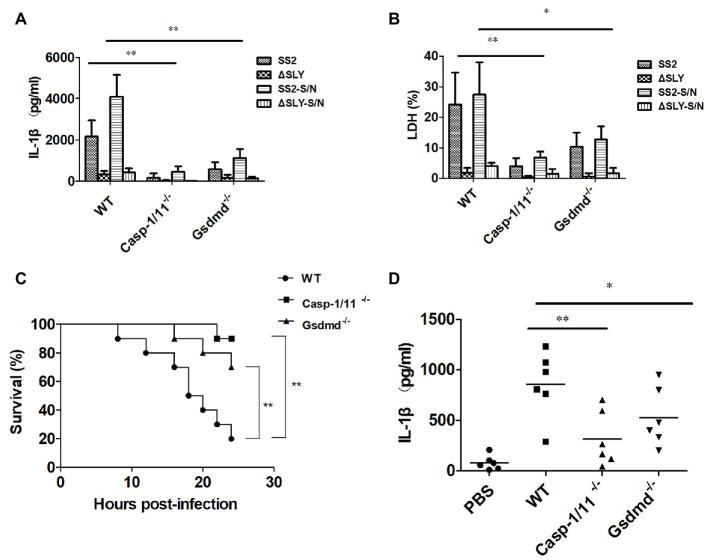
The SS2-induced IL-1β secretion was dependent on Caspase-1/11 and gasdermin D (Gsdmd) *in vitro* and *in vivo*. (**A**,**B**) BMDMs were isolated from 8-week-old WT C57BL/6J, *Caspase1/11*-deficient (Caspase-1/11^−/−^) and *Gsdmd*-deficient (Gsdmd^−/−^) mice, which were treated with SS2 (MOI, 1), ∆SLY (MOI, 1), SS2-S/N (200 ng/ml), and ∆SLY-S/N (200 ng/ml) for 16 h (LPS priming for 3 h was needed prior to the treatments of SS2-S/N and ∆SLY-S/N). The supernatants were collected for the IL-1β ELISA **(A)** and LDH assays **(B)**. **(C)** 8-week-old WT C57BL/6J, Caspase-1/11^−/−^, and Gsdmd^−/−^ mice were injected IP with a higher dose of SS2 (2 × 10^9^ CFU in 200 μl PBS) and monitored for 24 h for their survival, with 10 mice per group. The WT mice infected with SS2 were considered as the positive control group. **(D)** 8-week-old WT C57BL/6J, Caspase-1/11^−/−^, and Gsdmd^−/−^ mice were injected IP with a lower dose of SS2 (2 × 10^8^ CFU in 200 μl PBS). The wild-type (WT) mice infected with SS2 and PBS were considered as the positive and negative control group respectively. These mice were sacrificed 24 h after injection and their PLFs were harvested for the measurement of IL-1β (six mice per group). The data in panels (**A**,**B**) are the means ± standard deviations from three independent experiments. The data in panels (**C**,**D**) are from one of two independent experiments. ^*^p < 0.05; ^**^p < 0.01.

To verify the role of Caspase-1 and Gsdmd during SS2-induced-IL-1β release and the cytotoxicity in macrophages, we treated BMDMs isolated from WT, Caspase-1/11^−/−^ and Gsdmd^−/−^ mice with SS2, ∆SLY, SS2-S/N, and ∆SLY-S/N. The data showed the SS2 and SS2-S/N that induced IL-1β and the cytotoxicity were abolished in BMDMs isolated from Caspase-1/11^−/−^ mice, but partly inhibited in BMDMs from Gsdmd^−/−^ mice, compared with the cells of WT mice (Figures 3A,B).

To confirm the roles of Caspase-1/11 and Gsdmd in the pathogenicity of SS2-induced STSS *in vivo*, we infected IP WT, Caspase-1/11^−/−^, and Gsdmd^−/−^ mice with a higher dose of 2 × 10^9^ CFU SS2 and observed their activity and survival every 2 h until 24 h post infection. Three (3/10) Gsdmd^−/−^ mice and one (1/10) Caspase-1-deficient mouse died within 24 h post infection, and the deaths all decreased significantly compared to those of WT mice (8/10 died; Figure 3C).

To further evaluate whether Caspase-1/11 and Gsdmd were involved in the IL-1β release in response to the SS2 infection *in vivo*, we also injected IP WT, Caspase-1/11^−/−^, and Gsdmd^−/−^ mice with lower dose of 2 × 10^8^ CFU SS2. Our results showed that the IL-1β production in the PLF from SS2-infected WT mice was significantly higher than that in Caspase-1/11^−/−^ mice and Gsdmd^−/−^ mice (Figure 3D).

Thus, we confirmed that the activation of Caspase-1/11 and Gsdmd was required for the SS2-induced IL-1β release and cytotoxicity in BMDMs *ex vivo*, and this activation contributes to mouse death and IL-1β release in response to the SS2 infection *in vivo*.

### SS2-Triggered Caspase-1 Activation *via* the Nlrp3 Inflammasome, but Not the Nlrc4 and Aim2 Inflammasome

**Figure 4:**
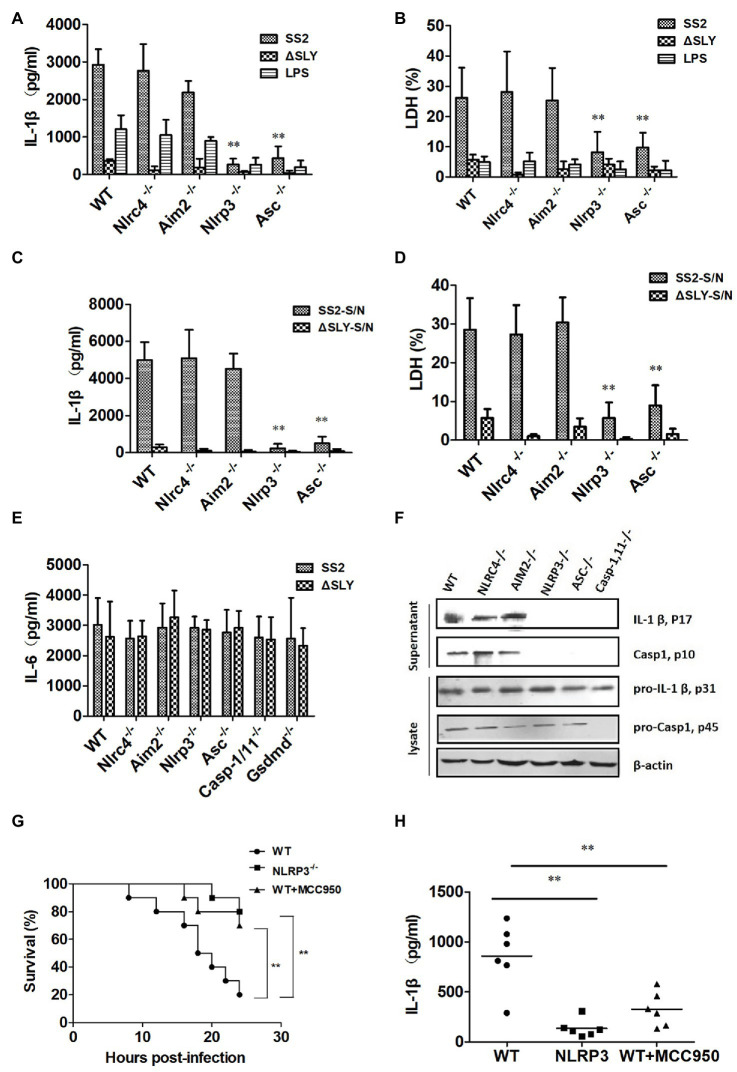
The SS2-induced IL-1β secretion is dependent on the Nlrp3 inflammasome activation *in vitro* and *in vivo*. BMDMs were isolated from WT C57BL/6J mice and multiple deficient mice [*Nlrc4*-deficient (Nlrc4^−/−^), *Aim2*-deficient (Aim2^−/−^), *Nlrp3*-deficient (Nlrp3^−/−^), and *Asc*-deficient (Asc^−/−^)] and infected with SS2 (MOI, 1) and ∆SLY (MOI, 1) for 16 h. The supernatants were harvested and assayed for IL-1β **(A)**, LDH **(B)**, and IL-6 **(E)**. These cells were also treated parallel with SS2-S/N (200 ng/ml) and ∆SLY-S/N (200 ng/ml) for 16 h after LPS priming for 3 h. Their supernatants were collected for IL-1β ELISA **(C)** and LDH assays **(D)**. **(F)** Along with immunoblotting, the supernatants were also measured for IL-1β p17 and Caspase1 p10, and the cell lysates were analyzed for pro-IL-1β p31 and pro-Caspase1 p45. **(G)** Three groups of 8-week-old mice, including WT mice (C57BL/6J), Nlrp3^−/−^ mice, and WT mice treated with the Nlrp3 inhibitor MCC950 (the WT mice were injected IP with Nlrp3 inhibitor MCC950 prior to SS2 infection), were injected IP with a higher dose of SS2 (2 × 10^9^ CFU in 200 μl PBS), and their survival was monitored for 24 h, with 10 mice per group. The WT mice infected with SS2 were considered as the positive control group. **(H)** Three groups of the abovementioned mice were injected IP with a lower dose of SS2 (2 × 10^8^ CFU in 200 μl PBS). The WT mice infected with SS2 were considered as the positive control group. These mice were sacrificed 24 h after injection and their PLF was harvested and measured for IL-1β (six mice in each group). The data in panels **(A–E)** are the means ± standard deviations from three independent experiments. The data in panels **(F–H)** are obtained from one of two independent experiments. ^*^p < 0.05; ^**^p < 0.01.

To characterize the specific inflammasome involved in SS2-induced Caspase-1 activation, we infected BMDMs of WT, Nlrp3^−/−^, Nlrc4^−/−^, Asc^−/−^, and Aim2^−/−^ mice with SS2, ∆SLY, SS2-S/N, and ∆SLY-S/N and measured the IL-1β release and cytotoxicity. The SS2-induced IL-1β and the cytotoxicity were completely abrogated in the Nlrp3^−/−^ and Asc^−/−^ BMDMs but unaltered in the Nlrc4^−/−^ and Aim2^−/−^ BMDMs (Figures 4A,B). Similarly, SS2-S/N induced significantly a greater amount of IL-1β and cytotoxicity in the BMDMs of WT, Nlrc4^−/−^, and Aim2^−/−^ BMDMs compared with those in the BMDMs of the Nlrp3^−/−^ and Asc^−/−^ mice (Figures 4C,D). In contrast, the IL-6 secretions from different genotypes of BMDMs were comparable, suggesting that Nlrp3 and Asc were dispensable for the production of IL-6 in response to the SS2 and ∆SLY strains (Figure 4E). Thus, the SS2-induced IL-1β secretions depend primarily on the Nlrp3 inflammasome activation.

We then measured the amount of inactive pro-IL-1β (p31), pro-Caspase-1 (p45), mature active IL-1β (p17), and Caspase-1 (p10) in the supernatants and cell lysates in SS2-infected-BMDMs from the WT and various deficient mice with immunoblotting. Consistent with the ELISA results, SS2 induced significantly more active IL-1β and Caspase-1 in the supernatants of the BMDMs of the WT, Nlrc4^−/−^, and Aim2^−/−^ mice but not in the BMDMs isolated from the Nlrp3^−/−^ and Asc^−/−^ mice. However, SS2 induced similar levels of biologically inactive pro-IL-1β and pro-Caspase-1 in cell lysates from various cells (Figure 4F). Thus, we demonstrated that the Nlrp3 inflammasome mediated the SS2-induced IL-1β release.

To further clarify the role of the Nlrp3 inflammasome activation in developing STSS caused by SS2, we infected three genotypes of mice [WT mice, Nlrp3^−/−^ mice, and WT mice injected with the Nlrp3 inhibitor (MCC950) prior to infection] with a high dose of 2 × 10^9^ CFU SS2. The results showed that the survival of the Nlrp3^−/−^ group (2 of 10 died) and WT mice treated with the inhibitor group (3 of 10 died) was both significantly higher than that of the WT group (8 of 10 died; Figure 4G). Therefore, we confirmed that the Nlrp3 inflammasome played a crucial role in the host’s combat against SS2 infection *in vivo*.

To further evaluate the role of the Nlrp3 inflammasome in the IL-1β release in response to the SS2 infection *in vivo*, we also injected IP three groups of the abovementioned mice with a lower dose of 2 × 10^8^ CFU SS2. The results showed that the IL-1β production in the PLF from the SS2-infected WT mice was significantly higher than that in the Nlrp3^−/−^ mice and WT mice injected with the Nlrp3 inhibitor (Figure 4H).

### SS2 and SS2-S/N-Induced IL-1β Release in BMDMs Requires K^+^ Efflux and ROS Production

**Figure 5:**
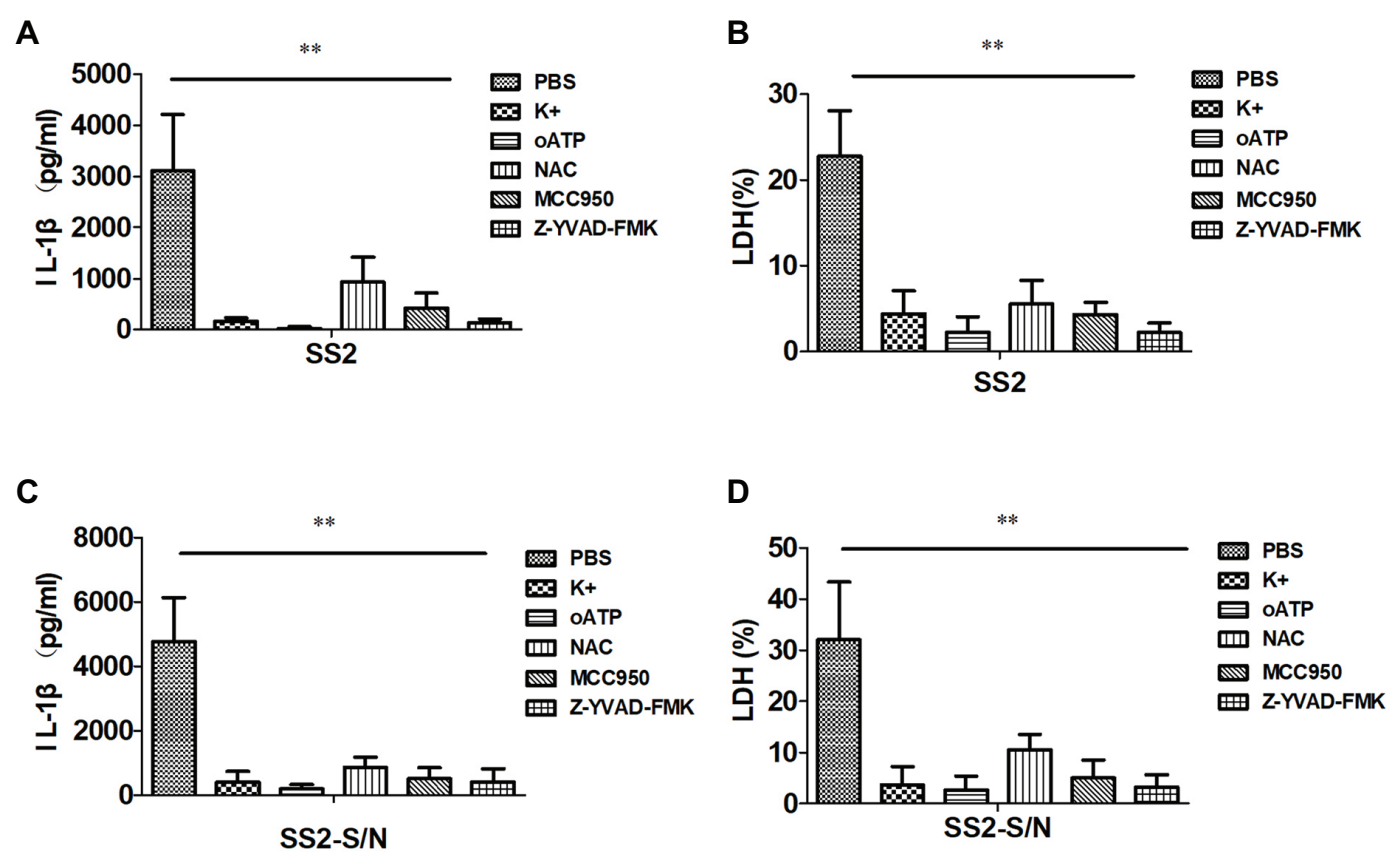
SS2-induced IL-1β secretion in BMDMs requires potassium (K^+^) efflux, ATP, and reactive oxygen species (ROS) production. BMDMs were infected with SS2 (MOI, 1) 16 h in the absence or presence of the K^+^ efflux blocker (KCl, 50 mM), ATP blocker (oxidized ATP, oATP, 500 μM), ROS inhibitor (N-acetyl-L-cysteine, NAC, 20 mM), Nlrp3 inhibitor (MCC950, 10 μM), and Caspase-1 inhibitor (Z-YVAD-FMK, 10 μM). IL-1β in the supernatants was measured with ELISA **(A)** and LDH was assayed using a Cytotox96 kit **(B)**. BMDMs were also incubated with SS2-S/N 16 h after lipopolysaccharide (LPS) priming for 3 h in the absence or presence of the above-mentioned inhibitors. The IL-1β in the supernatants was measured with ELISA **(C)**, and LDH was assayed using a Cytotox96 Kit **(D)**. The results are represented as the means ± standard deviations of three independent experiments. ^*^p < 0.05; ^**^p < 0.01.

To find whether potassium (K^+^) efflux participates in the inflammasome activation induced by SS2 in BMDMs, a high concentration of KCl was added to the supernatants of a cell culture to block the K^+^ efflux before the cells were treated with SS2. The IL-1β production and cytotoxicity were almost completely abrogated by KCl (Figures 5A,B). To investigate whether SS2 activated the inflammasome *via* the ATP receptor P2X7R, BMDMs were pretreated with the P2X7R inhibitor (oATP) prior to the SS2 infection. The results showed that the IL-1β release and cytotoxicity were significantly reduced by blocking the ATP receptor P2X7R (Figures 5A,B). To study the involvement of the ROS production in activating the inflammasome in response to the SS2 infection, BMDMs were pretreated with the ROS inhibitor (NAC). The IL-1β release and cytotoxicity triggered by SS2 was impaired by inhibiting ROS (Figures 5A,B). Together, these results indicated that the K^+^ efflux, ATP receptor P2X7, and production of reactive oxygen species (ROS) were all involved in IL-1β secretion during the SS2 infection. Furthermore, we pretreated BMDMs with the Nlrp3 inhibitor (MCC950) and Caspase-1 inhibitor (Z-YVAD-FMK) prior to the infection with SS2, and we observed the expected significant inhibitions to the IL-1β release and cytotoxicity (Figures 5A,B).

Similarly, the abovementioned five kinds of inhibitors, including KCl, P2X7R receptor inhibitor (oATP), ROS inhibitor (NAC), Nlrp3 inhibitor (MCC950), and Caspase-1 inhibitor (Z-YVAD-FMK), impaired the IL-1β release and cytotoxicity induced by SS2-S/N (Figures 5C,D).

All of the data suggested that the IL-1β secretion in BMDMs induced by SS2 or SS2-S/N was mediated by K^+^ efflux and ROS production *via* the Nlrp3 inflammasome activation.

## Discussion

Innate immunity is critical to the pathogenesis of the SS2 infection. The infected individuals rely exclusively on their innate immune response to clear bacteria but the massive release of proinflammatory mediators by innate immune cells can cause severe pathophysiological injury to the host (Li et al., 2019). In this study, we focused on the role of SLY and inflammasome activation in the pathogenesis of STSS-induced by SS2. Our results demonstrated that SLY mediated IL-1β release and cytotoxicity in the BMDMs, which contributed to the higher mortality of mice infected with SS2. We also found that the activation of the Nlrp3 inflammasome mediated by SLY played a key role in the IL-1β release, cytotoxicity, and protecting mice from early death due to the high dose of the SS2 infection in a mouse STSS model.

In this study, we proved that SLY, a member of cholesterol-dependent cytolysins, contributed to the IL-1β release, cytotoxicity, and activation of the Nlrp3 inflammasome that was involved in STSS caused by SS2. Our results add SLY to the growing list of bacterial toxins, including various pore-forming toxins, hemolysins, and cholesterol-dependent cytolysins, which can trigger the Nlrp3 inflammasome activation (Muñoz-Planillo et al., 2009; Meixenberger et al., 2010). SLY was also studied as a potential vaccine candidate (Du et al., 2013) because the increased SLY release promotes *S. suis* entering into and surviving in the blood stream, thereby contributing to the infection (Jacobs et al., 1996). Therefore, SLY plays a key role in the pathogenesis of SS2-induced STSS.

It has been reported that both Caspase-1‐ and Caspase-11-deficient mice are more resistant to LPS-induced shock than WT mice controls (Wang et al., 1998). SS2 is a gram-positive bacterium without LPS to activate Caspase-11. Therefore, we speculated that the STSS and pyroptosis induced by SS2 were mainly attributed to the canonical inflammatory Caspase pathway. Two studies reported that Gsdmd as a key mediator of pyroptosis was essential for IL-1β secretion in both the canonical‐ and non-canonical inflammatory Caspase pathways (He et al., 2015; Shi et al., 2015). The 31 kDa N-terminal fragment of Gsdmd is cytotoxic and capable of triggering pyroptosis when expressed aberrantly. Gsdmd-deficient macrophages failed to induce pyroptosis after exposure to cytosolic LPS and other known inflammasome stimuli, and therefore, Gsdmd was regarded as a shared component of Caspase-1‐ and -11-mediated pyroptosis. Whether Gsdmd is involved in the development of STSS in SS2 infection is uncertain. In this study, SS2-infected BMDMs from Caspase-1/11^−/−^and Gsdmd^−/−^ mice had a lower IL-1β release and cytotoxicity than the BMDMs from WT mice *ex vivo*. *In vivo* results further confirmed that Caspase-1/11^−/−^ mice and Gsdmd^−/−^ mice had a lower IL-1β production in the PLF and were more resistant to SS2-induced STSS and death compared with WT mice. These findings suggest that IL-1β release and pyroptosis mediated by activations of Caspase-1/11 and Gsdmd contribute to STSS during SS2 infection. Recent studies (Antonopoulos et al., 2015; Gurung and Kanneganti, 2015) revealed novel non-apoptotic function of caspase-8 to modulate IL-1β production *via* NLRP3 inflammasomes activation. It is proposed that caspase-8 regulates expression of pro-IL-1β and NLRP3 mRNA activation *via* nuclear factor-kB (NF-kB) pathway. In the NLRP3 inflammasome, caspase-8 is required for both canonical‐ and non-canonical assembly and activation of the inflammasome complex. In the absence of caspase-1, NLRP3 inflammasomes directly utilize caspase-8 as both a proapoptotic initiator and major IL-1β-converting protease. In the presence of caspase-1, caspase-8 acts as a positive modulator of the NLRP3-dependent caspase-1 signaling cascades that drive both IL-1β production and pyroptotic death. It remains to be determined whether caspase-8 is involved in SLY-induced NLRP3 activation and IL-1β production during SS2 infection.

Several types of streptococci have been reported to activate inflammasomes. For example, production of IL-1β in macrophages infected with *Streptococcus pyogenes* depends on the TLR and Nlrp3 signaling and the activation of Caspase-1 is mediated by the pore-forming toxin streptolysin O (Harder et al., 2009). Furthermore, Nlrp3 activation depends on pneumolysin, which is required for the protection against respiratory infections with *S. pneumonia* (McNeela et al., 2010). Moreover, the IL-1β secretion in Group B *Streptococcus*-stimulated mouse dendritic cells depended on the Nlrp3 inflammasome and on production of β-hemolysin by Group B *Streptococcus* (Costa et al., 2012). Recent studies indicated that *Streptococcus sanguinis* could trigger the IL-1β release *via* Nlrp3 inflammasome in macrophage and dendritic cells and that interaction of purinergic receptors with ATP released is involved in the activity (Saeki et al., 2017). How SS2 activates inflammasomes has not been well studied.

Coll et al. (2015) found that a small-molecule inhibitor, MCC950, showed its potential as a therapeutic drug in many NLRP3-associated syndromes, including autoinflammatory and autoimmune diseases. MCC950 specifically inhibits NLRP3 activation but not AIM2, NLRC4, or NLRP1 activation, and it blocks both canonical‐ and non-canonical inflammasome activation at nanomolar concentrations. Here, we reported that SS2-infected BMDMs from Nlrp3^−/−^ mice had a lower IL-1β release and cytotoxicity *ex vivo*. Both Nlrp3^−/−^ mice and WT mice treated with the Nlrp3 inhibitor MCC950 had a lower IL-1β production in the PLF and were more resistant to STSS and death caused by SS2 in comparison to the WT mice. Consistent with our results, Li et al. (2019) reported that myricetin, a natural compound, could reduce the production of the proinflammatory cytokines TNF-α and IL-1β effectively, and confirmed that myricetin exerted an obvious protective effect against SS2 infection *in vitro* and *in vivo*. Similarly, Shen et al. (2018) chose a mentoflavone, a biflavonoid compound that has been widely used in traditional Chinese medicine, to block SLY oligomerization and inhibit its pore-forming activity and suppress the excessive inflammation without interfering with SS2 growth, which were observed to clearly increase the survival of mice infected with highly virulent SS2. In contrast, Lavagna et al. (2019) reported that the survival of IL-1β receptor-deficient mice decreased compared to that of the WT mice when infected with 1 × 10^7^ CFU highly virulent SS2. The infection doses in Lavagna’s study were significantly lower than those in our study (2 × 10^9^ CFU) and Shen’s studies (2 × 10^9^ CFU; Shen et al., 2018). Besides the virulence of the pathogen and the genetic characters of the host, the infection dose of the pathogen may be another important risk factor that can induce an excessive inflammatory response and cause a severe outcome from the infection. We compared different infection doses and found that a higher infection dose can quickly induce an acute excessive inflammatory response as a cytokine storm and cause septic shock and death within 24 h. However, mice infected with a lower infection dose almost all survived. The inflammatory response to infection is beneficial to the host to combat bacterial growth. However, the massive release of proinflammatory mediators may cause severe pathophysiological phenomena that are the hallmark of SS2-induced septic shock and lethality. Therefore, reducing an excessive inflammatory response by using the SLY antibody or NLPR3 pathway inhibitor at an early stage may protect the host from severe acute STSS but might not play protective role at any stage of infection for any host with a different immune function.

The Nlrp3 inflammasome activation can be induced by diverse stimuli, including bacterial toxins and endogenous danger signals. We still do not know how the activation of the Nlrp3 inflammasome was initiated in the BMDMs infected with SS2. The K^+^ efflux occurs upon the extracellular ATP engagement with the ATP receptor P2X7R. Bacterial pore-forming toxins can cause plasma membranes to become more permeable, allowing the K^+^ efflux to be independent of the ATP receptor (Muñoz-Planillo et al., 2013). In this study, the IL-1β release in BMDMs was significantly reduced *via* blocking the K^+^ efflux and A2X7R receptor and inhibiting ROS generation. The roles of blocking the K^+^ efflux and A2X7R receptor were more effective than the inhibition of the ROS generation, suggesting that the K^+^ efflux may play the most important role in the SS2-induced IL-1β release. Lavagna et al. (2019) also reported consistent findings on the role of the K^+^ efflux in the Nlrp3 activation induced by SS2, although they also observed the activation of the NLRP1, Nlrc4, and Aim2 inflammasomes; Nlrp3 was the major one being activated (Mariathasan et al., 2006; Rathinam et al., 2010). The precise mechanism of the Nlrp3 inflammasome activation induced by SS2 should be further investigated.

In summary, we reported that SLY was essential for mediating the IL-1β secretion and triggering pyroptosis during the SS2 infection *in vitro* and *in vivo*. Our data suggested that Nlrp3 inflammasome activation and pyroptosis participated in the proinflammatory response upon SS2 infection. Furthermore, the K^+^ efflux and ROS generation are involved in the SLY-induced Nlrp3 inflammasome activation. Our study sheds light on the role of SLY and the activation of Nlrp3 inflammasomes and pyroptosis in the pathogenesis of STSS caused by the SS2 infection, and this may hold promise for the potential prevention and therapy for STSS.

## Data Availability Statement

The raw data supporting the conclusions of this article will be made available by the authors, without undue reservation.

## Ethics Statement

The animal study was reviewed and approved by the Laboratory Animal Welfare & Ethics Committee of the National Institute for Communicable Disease Control and Prevention, Chinese Center for Disease Prevention and Control.

## Author Contributions

LS and ZR conceptualized the experiments. LS, XL, YH, and YX conducted the experiments. LS analyzed the data. LS and ZR wrote the paper. YJ provided the strains and GM provided the deficient mice and both contributed to the discussion. All authors contributed to the article and approved the submitted version.

### Conflict of Interest

The authors declare that the research was conducted in the absence of any commercial or financial relationships that could be construed as a potential conflict of interest.

## Abbreviations

SLY: suilysin
SS2: *Streptococcus suis* serotype 2
STSS: streptococcal toxic-shock-like syndrome
NLRs: NOD-like receptors
Asc: adapter molecule
Nlrp3: NLR family pyrin domain containing 3
ROS: reactive oxygen species
WT: wild-type
∆SLY: isogenic *sly* mutant
PLF: peritoneal lavage fluid
Asc^−/−^: Asc-deficient
Nlrp3^−/−^: Nlrp3-deficient
Casp-1/11^−/−^: Caspase-1/11-deficient
BMDMs: mouse bone marrow derived macrophages
PMs: mouse peritoneal macrophages
IP: intraperitoneally
MOI: multiplicity of infection
LDH: lactate dehydrogenase
Gsdmd: gasdermin D
